# Intelligent prediction of lower extremity loadings during badminton lunge footwork in a lab-simulated court

**DOI:** 10.3389/fbioe.2023.1229574

**Published:** 2023-08-08

**Authors:** Lin Yu, Hanhui Jiang, Qichang Mei, Nur Ikhwan Mohamad, Justin Fernandez, Yaodong Gu

**Affiliations:** ^1^ Faculty of Sports Science, Ningbo University, Ningbo, China; ^2^ Research Academy of Grand Health, Ningbo University, Ningbo, China; ^3^ Auckland Bioengineering Institute, The University of Auckland, Auckland, New Zealand; ^4^ Faculty of Sports Sciences and Coaching, Sultan Idris Education University, Tanjong Malim, Malaysia; ^5^ Department of Engineering Science, The University of Auckland, Auckland, New Zealand

**Keywords:** lunging step, court sports, wearables, machine learning, knee, ankle

## Abstract

**Introduction:** Playing badminton has been reported with extensive health benefits, while main injuries were documented in the lower extremity. This study was aimed to investigate and predict the knee- and ankle-joint loadings of athletes who play badminton, with “gold standard” facilities. The axial impact acceleration from wearables would be used to predict joint moments and contact forces during sub-maximal and maximal lunge footwork.

**Methods:** A total of 25 badminton athletes participated in this study, following a previously established protocol of motion capture and musculoskeletal modelling techniques with the integration of a wearable inertial magnetic unit (IMU). We developed a principal component analysis (PCA) statistical model to extract features in the loading parameters and a multivariate partial least square regression (PLSR) machine learning model to correlate easily collected variables, such as the stance time, approaching velocity, and peak accelerations, with knee and ankle loading parameters (moments and contact forces).

**Results:** The key variances of joint loadings were observed from statistical principal component analysis modelling. The promising accuracy of the partial least square regression model using input parameters was observed with a prediction accuracy of 94.52%, while further sensitivity analysis found a single variable from the ankle inertial magnetic unit that could predict an acceptable range (93%) of patterns and magnitudes of the knee and ankle loadings.

**Conclusion:** The attachment of this single inertial magnetic unit sensor could be used to record and predict loading accumulation and distribution, and placement would exhibit less influence on the motions of the lower extremity. The intelligent prediction of loading patterns and accumulation could be integrated to design training and competition schemes in badminton or other court sports in a scientific manner, thus preventing fatigue, reducing loading-accumulation-related injury, and maximizing athletic performance.

## Key points:

• We developed principal component analysis (PCA) and partial least square regression (PLSR) statistical models to predict the knee- and ankle-joint loadings during badminton footwork from wearables.
• Flexion moment, AP, and axial contact forces in the knee were significantly higher during maximal lunges.
• During maximal lunges, dorsiflexion moment, AP, and axial contact forces in the ankle were significantly higher than those during sub-maximal lunges.
• Key variances (over 73%) between the vertical GRF of sub-maximal and maximal lunges were located in the initial and secondary impact peaks.
• Inertial magnetic unit (IMU) attached to the ankle showed promise (93%) to predict biomechanical loadings in the knee and ankle joints.

## 1 Introduction

Badminton lunges typically manifest as unilateral movements with the upper and lower extremities on the same side (depending on the right- or left-limb dominant) (Yu et al., 2021a). The current study followed up our previous study on the investigation of knee-joint loadings during directional badminton lunges using musculoskeletal-driven finite element modelling, which was supported by the Badminton World Federation (BWF) research project, reporting that the left-side (forecourt and backcourt) backhand lunges exhibited larger knee loadings (i.e., joint moments and contact forces) compared to the right-side (forecourt and backcourt) forehand lunges (Yu et al., 2021a). It was further proposed that the next-step research will focus on the dynamic monitoring of lower extremity loadings from the lab-simulated court toward on-court intelligent monitoring. A key issue observed in the previous studies was that the lab-simulated studies strictly controlled variables, which is not the case during real on-court training and competition.

Considering the high ratio of injuries in the lower extremity, particularly the injuries in the knee and ankle joints have been commonly reported and documented in previous studies, such as ankle sprains (fractures), knee pains, and Achilles tendon ruptures, among others (Chard and Lachmann, 1987; Jérgensen and Winge, 1987; Kroner et al., 1990; Fahlström et al., 1998; Fong et al., 2007). Lab-simulated experiments were conducted to reveal and understand the potential injury mechanism (Lee and Loh, 2019; Lam et al., 2020; Phomsoupha and Laffaye, 2020). However, it was also acknowledged that the biomechanical experiments conducted in the lab environment are different from “real” on-court training and competition.

The prevention of injuries in badminton has been an area of avid interest for sports scientist, biomechanist, physical therapist, and sport medicine clinician. Based on the contributing mechanism, the injuries have been classified as chronic injury due to the reason of repetitive loading accumulation and acute injury from unexpected incursion (Goh et al., 2013; Reeves et al., 2015). Our recent study has revealed the loading patterns of the knee joint from directional lunges in a lab-simulated court and reported a higher loading in the backhand side (Yu et al., 2021a). The challenge of discrepancy between lab tests and on-court situation was further reported and highlighted, and a badminton-specific task with wearable and adjustable loads was proposed to improve training specificity (Yu and Mohamad, 2022). Recently, the rapidly emerging wearable technology in the biomechanics research community provided plausible and accessible approaches to solving this challenging issue, with the integration of machine learning and artificial intelligence techniques. These have been implemented in the measurement of gait patterns (Shahabpoor and Pavic, 2017) and monitoring of running load accumulation (Ueberschär et al., 2019; Van Hooren et al., 2020).

The purpose of the current study was to conduct a perspective study toward the monitoring of loads in the knee and ankle joints using wearable technology and machine learning estimation. Thus, this study first correlated the data from wearables using the ground-truth lab test to develop and validate intelligent machine learning models. In particular, a PCA model was developed for feature extraction and dimensionality reduction, thus correlating data in wearables using the “ground-truth” lab test, and a PLSR machine learning model was used for multivariate correlation and prediction to estimate the loads in the “real” on-court badminton training and competition.

## 2 Methodology

### 2.1 Participants

A total of 25 experienced male badminton athletes (age: 24.3 ± 4.5 years; height: 175 ± 3.6 cm; weight: 71 ± 4.2 kg; years of badminton playing: 7.1 ± 3.2 years; all were right-hand dominant) participated in the lab test, and the synchronized collection of motion data and IMU data (fist session) was performed. This study was approved by the Ethical Committee of the Research Institute in Ningbo University (RAGH20190901). All athletes were informed of the requirements, objectives, and procedures of the lab and on-court tests, and written consent was obtained.

### 2.2 Protocol

**FIGURE 1:**
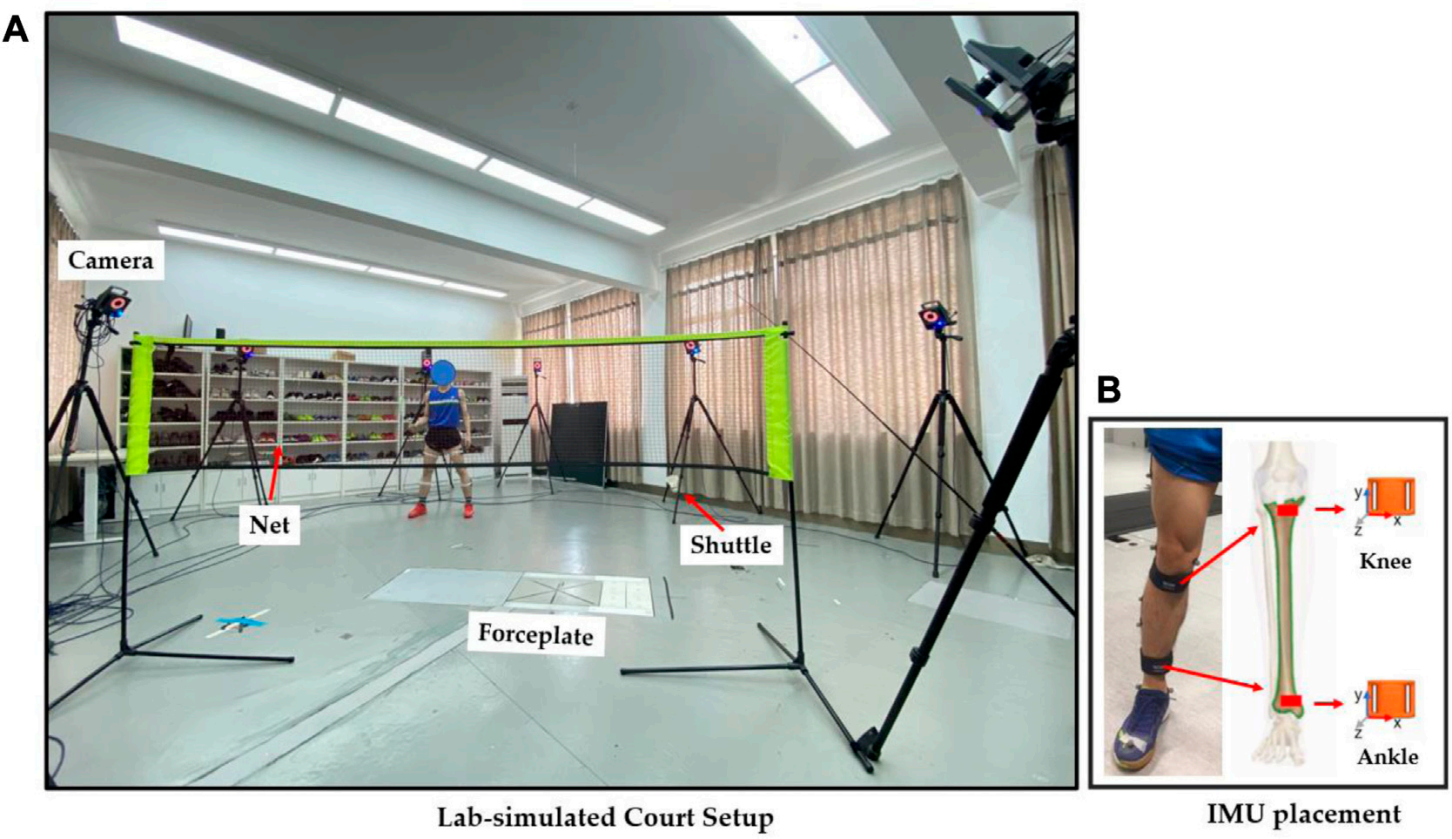
Illustration of the experimental setup **(A)** and IMU placement **(B)**.

The first session of the lab test synchronized the 3D motion capture and wearable sensors. The test involved a 12-camera Vicon system and Vicon IMU wearable sensors (Vicon Metrics Ltd., Oxford, United Kingdom) and an AMTI 3D force plate (AMTI, Watertown, MA, United States) (Figure 1). The collection frequency of the Vicon camera system was set at 200 Hz, and the IMU and force plate were set at 1000 Hz.

In order to mimic the real movements, we employed an established full-body marker-set model during motion capture (Rajagopal et al., 2016). The IMU sensors for the ankle and knee joints are illustrated in Figure 1B. In particular, the knee IMU was placed 2 cm below the medial condyle of the proximal tibia, and the ankle IMU was placed 3 cm above the medial malleolus of the distal tibia (Sheerin et al., 2020). The vertical axis of the IMU sensor was axially aligned with the midpoint of the ankle and knee joints in the tibia, which was validated in a clinical protocol of our previous study (Yeung et al., 2022). To follow up on our previous studies (Mei et al., 2017; Yu et al., 2021a), the four directional sub-maximal and maximal lunges were performed with a synchronous collection of motion, ground reaction force, and IMU data for further processing and analysis. Particularly, the sub- and maximal-right-forward forehand lunges (***Sub-RF*** and ***Max-RF***), sub- and maximal-left-forward backhand lunges (***Sub-LF*** and ***Max-LF***), right-backward backhand sub-maximal and maximal lunges (***Sub-RB*** and ***Max-RB***), and left-backward backhand sub-maximal and maximal lunges (***Sub-LB*** and ***Max-RB***) were performed with 80% (for sub-maximal) and 100% (for maximal) efforts, following the previous protocol (Lam et al., 2017; Lam et al., 2018; Yu et al., 2021a).

### 2.3 Data processing

The joint kinematics, kinetics, and contact forces were calculated, following the previously established protocols of musculoskeletal OpenSim modelling. Machine learning models were also developed and tested using motion capture data against the acceleration and angular velocity data from wearable sensors.

**FIGURE 2:**
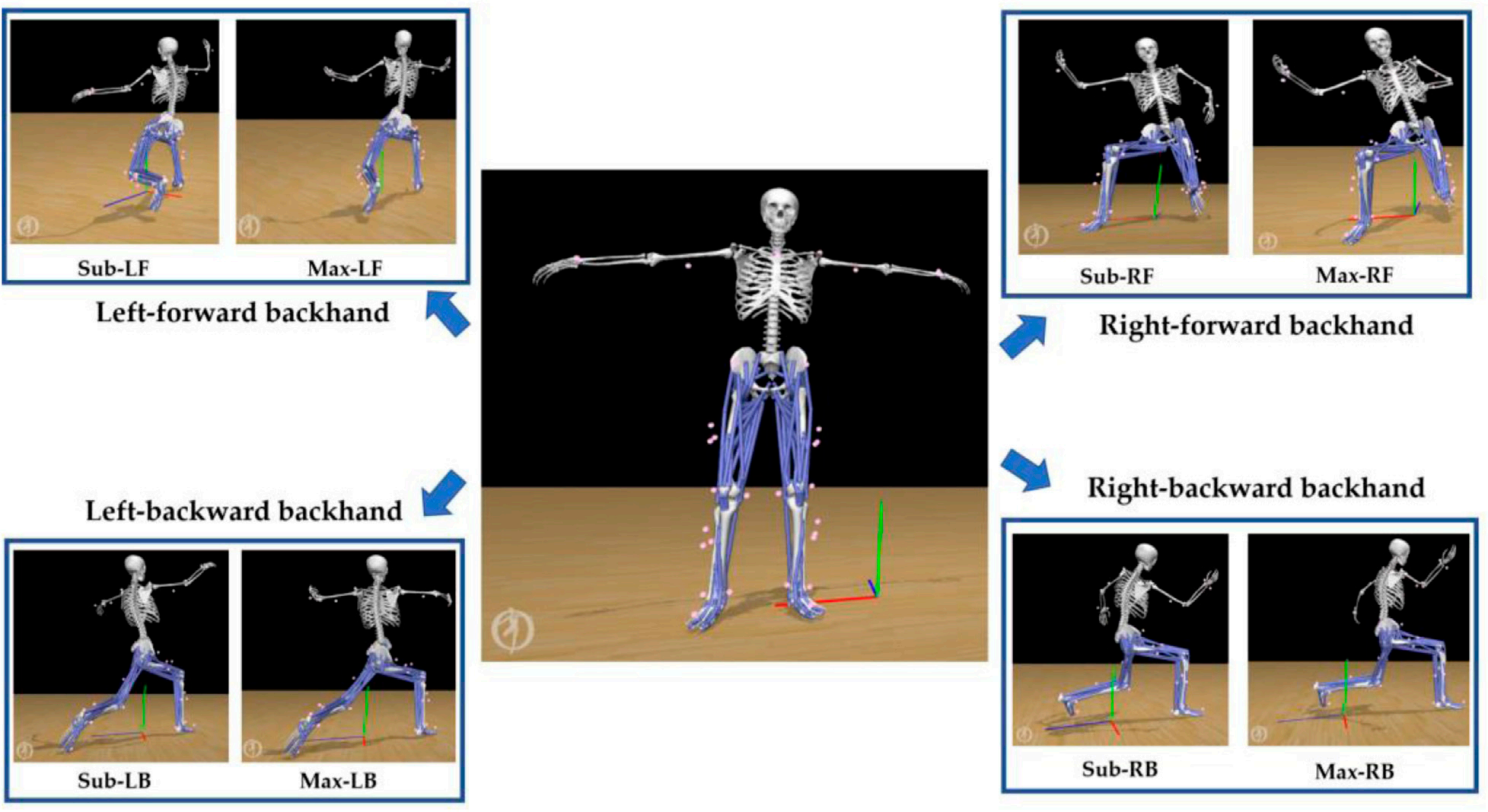
Illustration of the musculoskeletal modelling pipeline.

First, the static marker positions and body mass were used to “scale” the generic model to match subject-specific musculoskeletal models (Figure 2), as per the standardized workflow (Delp et al., 2007), which was validated in our recent studies (Mei et al., 2019; Yu et al., 2021a). The “inverse kinematics” (***IK***) algorithm, which minimized errors between virtual markers in the model and experimental marker trajectories, was applied to compute joint angles. Then, the “*inverse dynamics*” (***ID***) algorithm was performed to calculate joint moments, and the “*static optimization*” (***SO***) algorithm was applied to compute muscle activation and forces. The estimated muscle activation was compared with the measured surface EMG signals to validate the model (Yu et al., 2021a). Lastly, the contact forces towards the knee and ankle joints in the anterior/posterior (x), superior/inferior (y), and medial/lateral (z) directions were computed using “j*oint reaction*” (*JR*) analysis.

**FIGURE 3:**
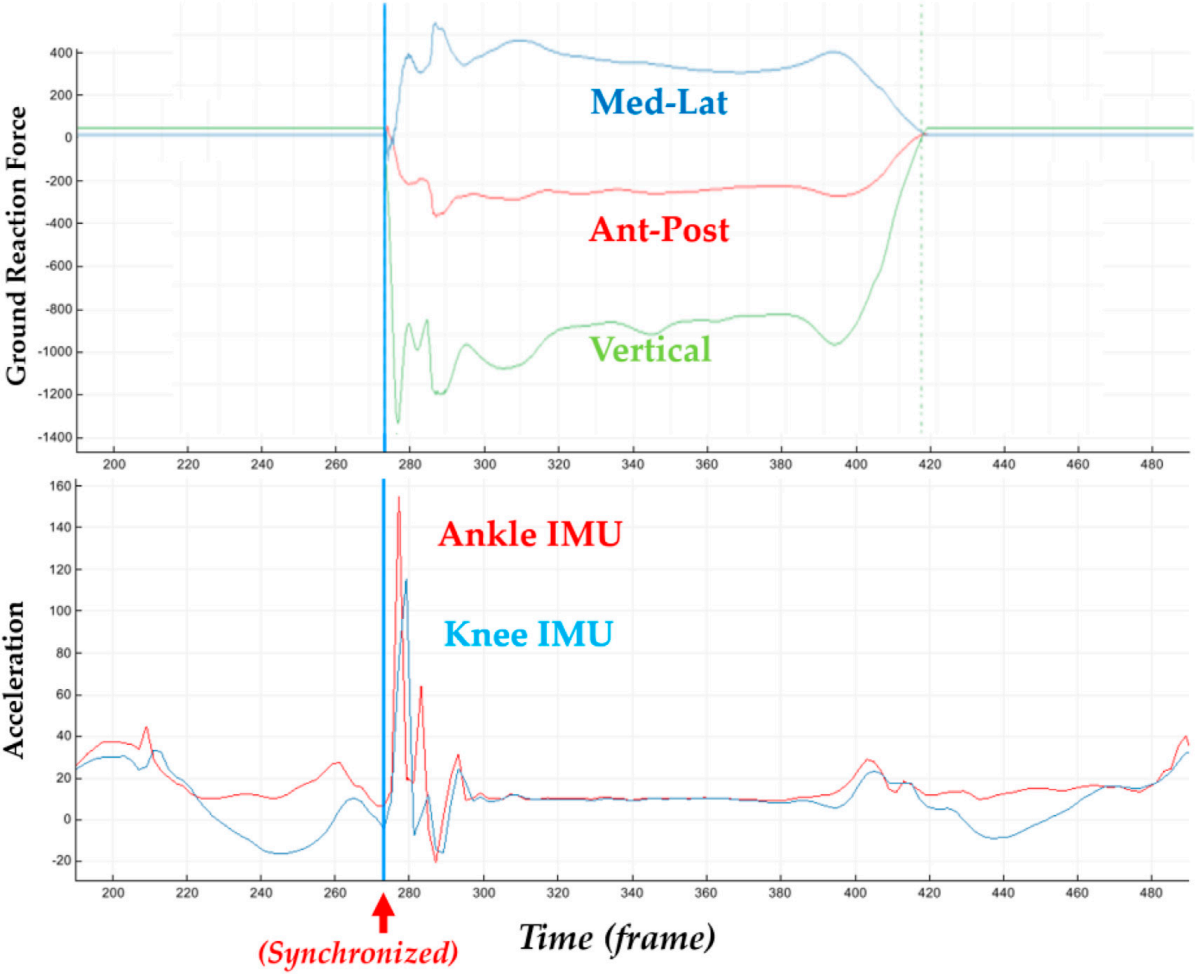
Illustration of synchronized ground reaction forces with IMU acceleration signals of the knee and ankle in the axial direction.

In addition to the biomechanical variables, we also calculated the parameters of contact time, approaching velocity, and peak IMU accelerations (G) in the knee and ankle joints, as shown in Figure 3. The loading rate was calculated following the previous established protocol (Mei et al., 2019; Yu et al., 2021b). The axial (**y**-axis) acceleration of particular interest was taken for analysis to quantify the accumulation of impact in the lower extremity (tibia) (Rice et al., 2019; Tenforde et al., 2020), which was normalized by gravitational acceleration (**G** = 9.8 m/s^2^).

**FIGURE 4:**
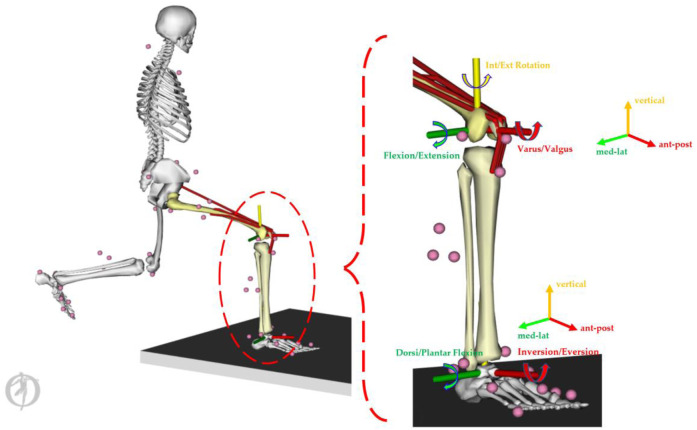
Illustration of the knee and ankle loading parameters.

The processed time-varying moment and contact force parameters during the lunging stance (Figure 4) were interpolated (normalized) into a 101 datapoint for statistical modelling (Yu et al., 2021b; Mei et al., 2021). Particularly, the biomechanical parameters included the knee flexion/extension moment, knee varus/valgus moment, knee int/ext rotation moment, ankle dorsi/plantar flexion moment, ankle inversion/eversion moment, knee ant-post/med-lat/vertical contact forces, and ankle ant-post/med-lat/vertical contact forces. The joint moment was normalized to body mass in kg (unit: Nm/kg), and the joint force was normalized to body weight (unit: times Newton in BW).

### 2.4 Statistical analysis and modelling

The discrete values of approaching velocity, stance time, knee and ankle acceleration, peak knee and ankle moments, and peak knee and ankle joint contact forces were first checked for the normality distribution and were compared using the paired sample *t*-test with a significance level at 0.05. The time-varying joint moment and force over stance were then modelled with the multivariate statistical models. In the current study, the statistical models were developed and validated, as described in our previous studies, using MATLAB software (R2019a, MathWorks Inc., MA, United States of America), specifically PCA (Yu et al., 2021b) and PLSR modelling (Mei et al., 2020).

In this study, the PCA multivariate technique (Wold et al., 1987; Lever et al., 2017; Yu et al., 2021b) was used to reduce the high-dimensional data matrices into orthogonal principal components (PCs), which explained major variations within the dataset (Deluzio et al., 1997; Lever et al., 2017). Each variation reported in the PCA modelling was a feature extraction applied in the machine learning (PLSR) technique (Phinyomark et al., 2018).

As presented in Eq. 1, the original matrices (*X = x* ^*1*^ *, x* ^*2*^ *, x* ^*3*^ *, …, x* ^*99*^ *, x* ^*100*^ *, x* ^*101*^) *m were orthogonally transformed into uncorrelated principal components (*Z = z* ^*1*^ *, z* ^*2*^ *, z* ^*3*^ *, … , z* ^*p*^) (*p* < 101), corresponding loading vectors (*T* ^*2*^ *= T*_*1*_ *, T*_*2*_ *, T*_*3*_ *, …, T*_*m*_), and residuals (*Q*), which was defined as Z = X*T ^*2*^ (Deluzio et al., 1997).

$$\left[\begin{array}{ccc} x_{1}^{1} & x_{1}^{2}\cdots & x_{1}^{100}\;x_{1}^{101}\\ \vdots & \vdots \ddots & \vdots \\ x_{m}^{1} & x_{m}^{2}\cdots & x_{m}^{100}\;x_{m}^{101} \end{array}\right]=\left[\begin{array}{ccc} z_{1}^{1} & z_{1}^{2} & z_{1}^{3}\\ \vdots & \vdots & \vdots \\ z_{m}^{1} & z_{m}^{2} & z_{m}^{3} \end{array}\right]\begin{array}{c} T_{1}^{2}\\ \vdots \\ T_{m}^{2} \end{array}\begin{array}{c} Q_{1}\\ \vdots \\ Q_{m} \end{array}.$$ (1)

The ***m*** equals 200 (4*2*101 matrices) for PCA modelling of the four lunges (RF, LF, RB, and LB) with sub-maximal and maximal performance. This study mainly considered the main variations in the first three PCs (***z*** ^***1***^ **, *z*** ^***2***^ **,** and ***z*** ^***3***^), which accounted for approximately 85–90% of the variation (Yu et al., 2021b). The variations in the vertical ground reaction force, knee and ankle moments, and contact forces of the first three PCs were then plotted against the mean for the visualization of the key features of variances, with “***+***” and “**▽**” representing the upper and lower limits, respectively.

In terms of PLSR statistical modelling (Wold et al., 1984; Mei et al., 2020), the two fundamental equations are the predictor matrix (***X***_***NM***_) and the response matrix (***Y***_***NP***_), which are expressed as follows:

$$X_{NM}=T_{NL}\;P_{ML}^{T}+E_{NM},$$ (2)

$$Y_{NP}=U_{NL}\;Q_{PL}^{T}+F_{NP}.$$ (3)

The subscript *N* represents the number of datasets (25*4 training samples in this study). The subscript *M* represents the number of predictor variables (four metrics, namely, contact times, velocity, peak knee G, and peak ankle G). The subscript ***P*** represents the number of response variables (12 loading variables, such as loading rate, knee flex-extension/varus-valgus/int-ext rotation moments, ankle dorsi-plantar flexion/inversion-eversion moments, knee ant-post/med-lat/vertical contact forces, and ankle ant-post/med-lat/vertical contact forces), and the subscript *L* represents the number of components.

*T* and *U* are the projection matrices (also called the scores); *P* and *Q* are the transposed orthogonal loading matrices (where the rows are created from eigenvectors or principal components); and *E* and *F* are the error or residual terms. The score vectors are related using a linear function, *U* = *f(T)* + *H*, where *H* is the vector of residuals.

## 3 Results

### 3.1 PCA

**FIGURE 5:**
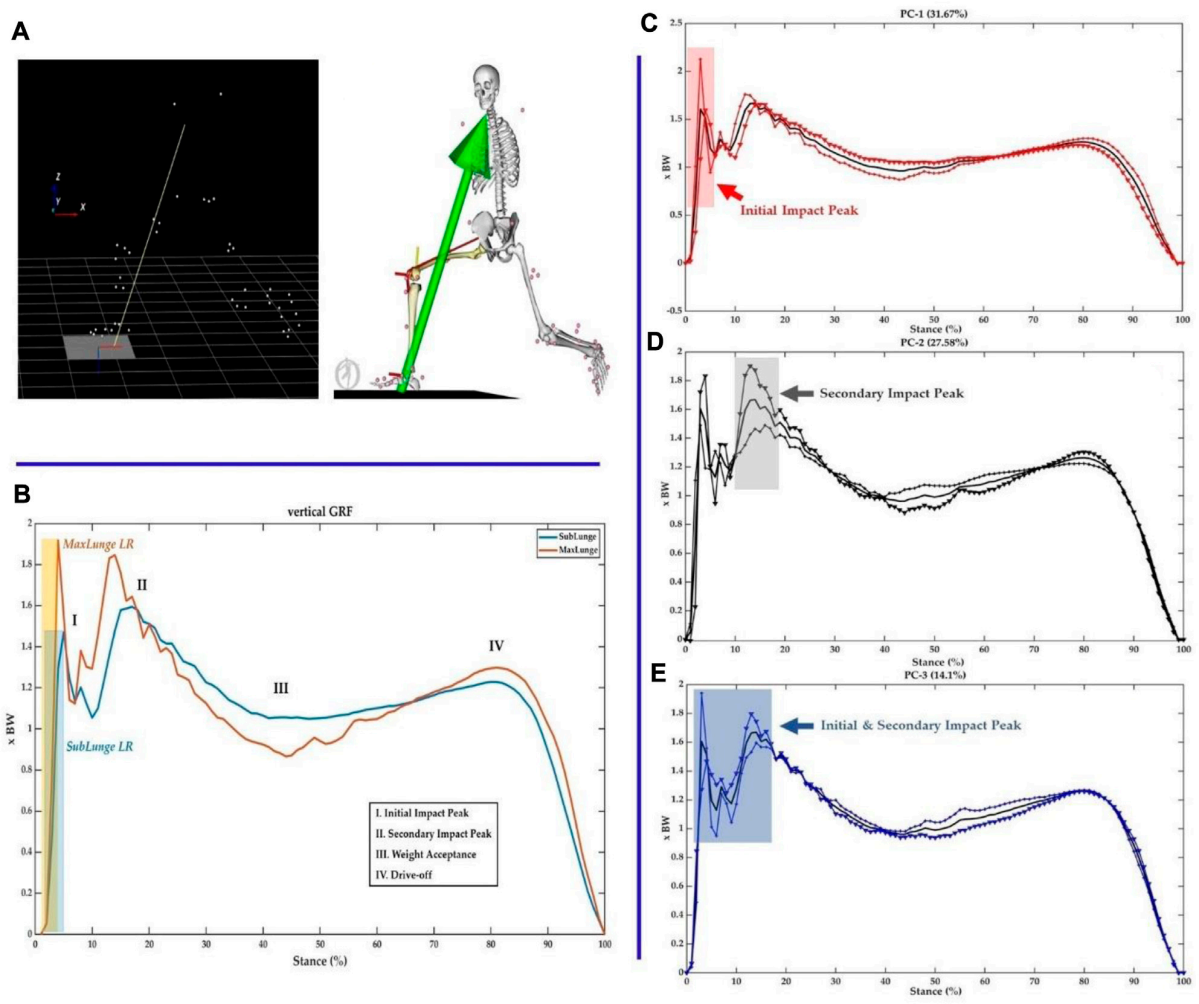
Illustration of data collection **(A)**, classification of four phased in the vertical ground reaction force during lunging **(B)** and three principal modes of variance **(C–E)**.

The vertical ground reaction force (Figure 5A) was classified into four key phases: initial impact peak (I), secondary impact peak (II), weight acceptance (III), and drive-off (IV) phases (Figure 5B). Following the PCA modelling of the vertical GRF, the first mode (PC1, Figure 5C) showed the main variations (percentage of variation explained: 31.67%) from landing to the initial impact peak (Phase-I), where the loading rate (LR) was calculated as a key impact parameter. The second mode (PC2, Figure 5D) occurred in the secondary impact peak (Phase-II) (27.58%), and the third mode (PC3, Figure 5E) was the combination of variations (14.1%) in both the initial and secondary impact peaks (Phase-I and Phase-II), which was the impact transient.

**FIGURE 6:**
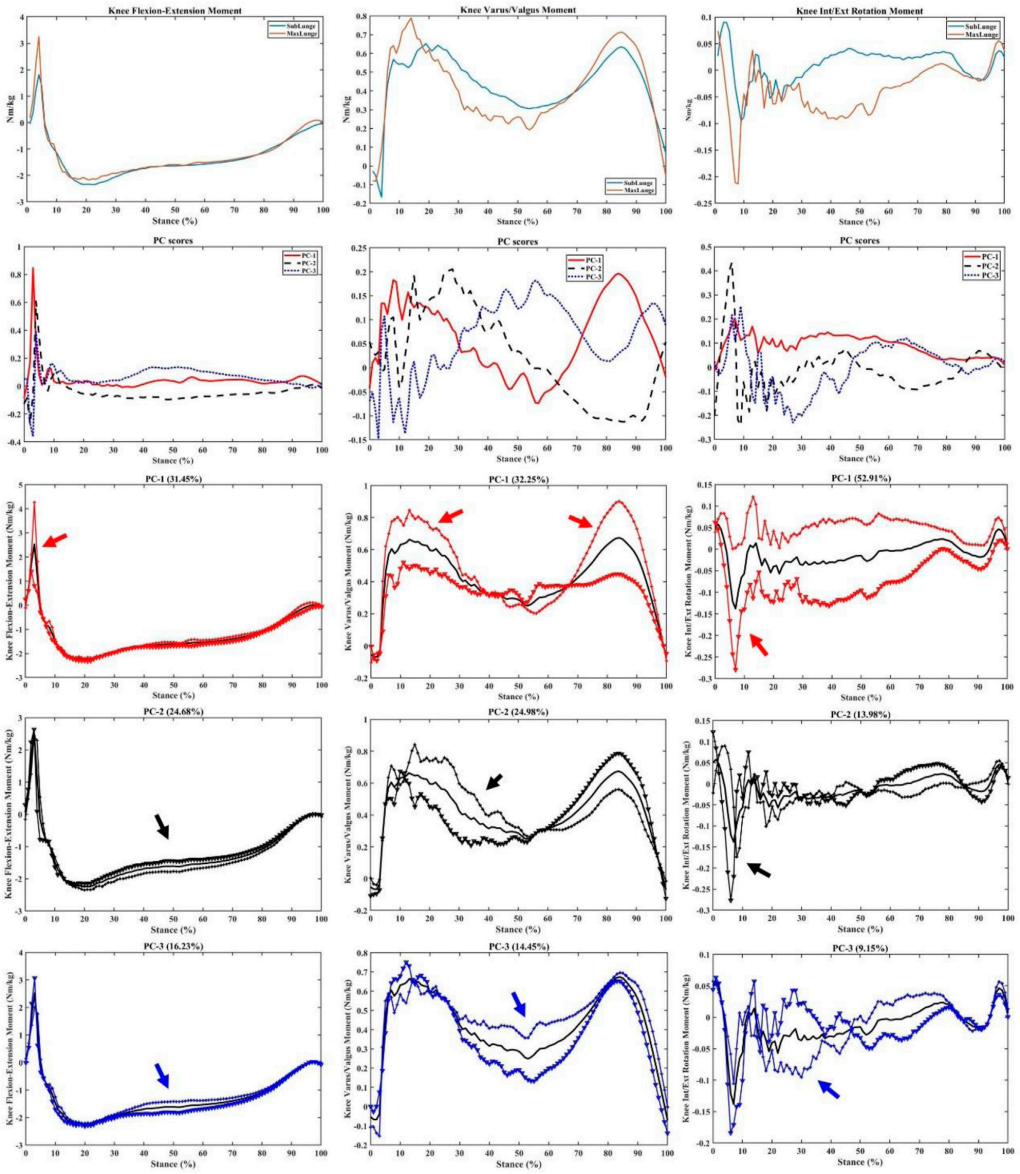
Knee moments (mean), PC scores, and key modes of variations (PC1, PC2, and PC3) against the mean with the illustration of the upper (+) and lower (▼) limits.

Consistent with the variations in vertical GRF, the knee flexion/extension, varus/valgus and int/ext rotation moments showed great variances mainly during the landing (impact absorption) phase. As a particular interest to illustrate key variations, the PC1 of knee flexion/extension (31.45%, impact phase), varus/valgus (32.25%, landing and drive-off phases), and int/ext rotation (52.91%, over the stance) moments is presented in Figure 6, with highlighted regions.

**FIGURE 7:**
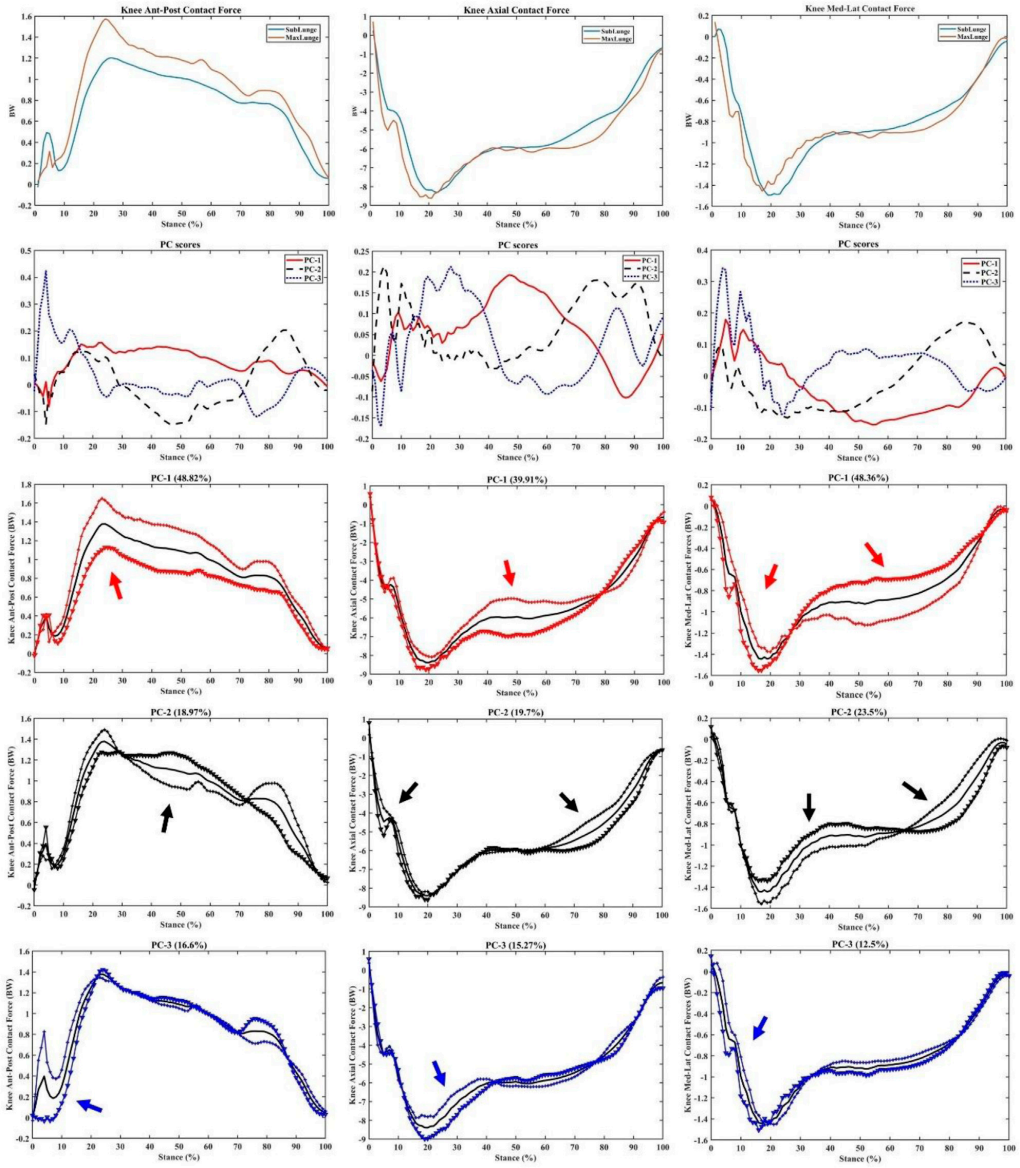
Knee forces (mean), PC scores, and key modes of variations (PC1, PC2, and PC3) against the mean with the illustration of the upper (+) and lower (▼) limits.

As shown in Figure 7, the key variations in the knee contact forces were observed over the stance with PC1 of ant-post (48.82%) and med-lat (48.36%) forces, while the axial contact force was mainly observed during the mid-stance (39.91%), especially the weight acceptance phase showing the difference between sub-maximal and maximal lunging steps.

**FIGURE 8:**
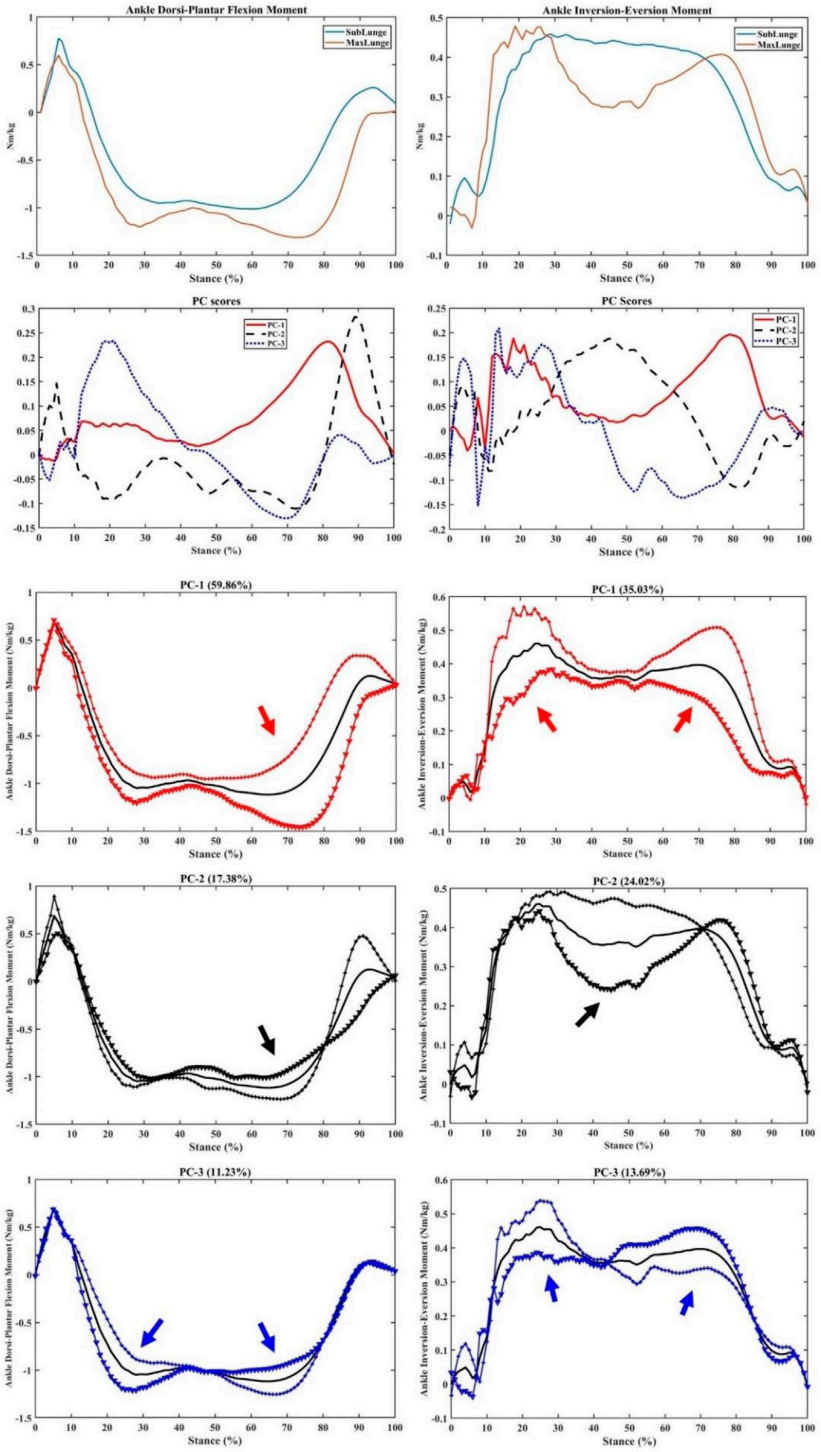
Ankle moments (mean), PC scores, and key modes of variations (PC1, PC2, and PC3) against the mean with the illustration of the upper (+) and lower (▼) limits.

The ankle dorsi/plantar flexion and inversion–eversion moments had a principal variance over the stance, especially PC1 (59.86% and 35.03%, respectively) during impact absorption and drive-off phases (Figure 8). It is worth noting the secondary variation in the dorsi/plantar flexion moment during the drive-off phase (PC2: 17.38%), which may explain the difference in ankle contributions during the push-off phase to the return phase. The inversion–eversion moments mainly varied during mid-stance (PC: 24.02%), which was another difference between sub-maximal and maximal lunges.

**FIGURE 9:**
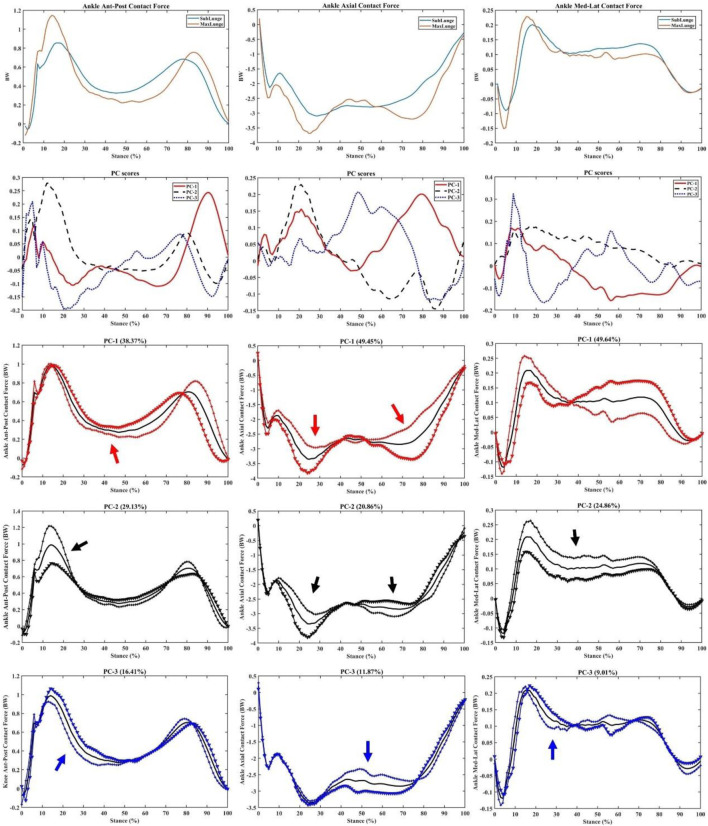
Ankle forces (mean), PC scores, and key modes of variations (PC1, PC2, and PC3) against the mean with the illustration of the upper (+) and lower (▼) limits.

Similarly for the ankle contact forces, great variations (PC1) in the impact peaks (initial and secondary) and drive-off phases were observed in the ant-post (38.37%), axial (49.45%), and med-lat (49.64%) vectors (Figure 9). Secondary (PC2) variations in the impact peaks were found in the ant-post (29.13%) and axial (20.86%) forces, while the med-lat forces varied across the stance (24.86%).

### 3.2 PLSR

Following PCA modelling, statistical comparisons of contact times, approaching velocities, peak acceleration (knee), and peak acceleration (ankle) were conducted, as presented in Table 1, which were then input as predictors in the PLSR machine learning model. Sub-maximal lunges showed shorter contact times, smaller approaching velocity, and smaller ankle impact than those in maximal lunges in four directions.

**TABLE 1:** Statistics of predictor metrics (time, velocity, knee impact, and ankle impact).

	Sub		Max		*p*
	Mean ± SD	95%CI	Mean ± SD	95%CI	
Time (s)	0.64 ± 0.06	0.59–0.68	0.71 ± 0.04	0.67–0.75	**0.02**
Velocity (m/s)	2.1 ± 0.15	1.96–2.21	2.7 ± 0.18	2.58–2.83	**<0.00**
Knee impact (G)	7.9 ± 2.44	5.72–10.24	7.6 ± 3.4	5.38–9.9	0.82
Ankle impact (G)	8.96 1.88	7.82–10.09	11.1 0.98	9.97–12.24	**0.01**

Bold values indicates significance.

*LR*, peak knee flexion/extension, varus/valgus, and int/ext rotation moments, peak ankle dorsi/plantar flexion and inver/eversion moments, peak knee ant-post/med-lat/axial (vertical) contact forces, and peak ankle ant-post/med-lat/axial (vertical) contact forces were analyzed, as shown in Table 2. The maximal lunges had greater LR, joint moments, and contact forces overall than those in submaximal lunges. These discrete parameters were then used as response metrics to train the PLSR machine learning model.

**TABLE 2:** Statistics of response metrics (loading rate and knee and ankle moments and forces).

			Sub		Max		*p*
			Mean ± SD	95%CI	Mean ± SD	95%CI	
LR (BW/s)			80.6 ± 16.4	60.92–100.29	133.4 ± 32.8	113.7–153.1	0.001
Knee (Nm/kg) (*BW)	Flexion moment		2.1 ± 0.15	1.96–2.21	2.7 ± 0.18	2.58–2.83	**<0.000**
	VV moment		7.9 ± 2.44	5.72–10.24	7.6 ± 3.4	5.38–9.9	0.82
	Int/ext rot moment		8.96 ± 1.88	7.82–10.09	11.1 ± 0.98	9.97–12.24	**0.01**
	AP force		1.35 ± 0.19	1.19–1.51	1.92 ± 0.22	1.76–2.08	**<0.000**
	Axial force		9.4 ± 0.82	8.19–10.61	11.37 ± 2.1	10.16–12.58	**0.026**
	ML force		1.84 ± 0.17	1.61–2.07	2.37 ± 0.4	2.15–2.61	**0.003**
Ankle (Nm/kg) (*BW)	Dorsi/plantar-flexion moment		1.22 ± 0.08	0.97–1.48	1.79 ± 0.47	1.54–2.05	**0.0043**
	Inv/eve moment		0.74 ± 0.27	0.2–1.48	1.73 ± 1.34	0.99–2.46	0.061
	AP force		1.04 ± 0.12	0.88–1.2	1.5 ± 0.27	1.34–1.66	**0.0006**
	Axial force		3.41 ± 0.23	3.03–3.79	4.59 ± 0.67	4.21–4.97	**0.0004**
	ML force		0.29 ± 0.05	0.24–0.35	0.47 ± 0.09	0.41–0.53	0.4

Bold values indicates significance.

**FIGURE 10:**
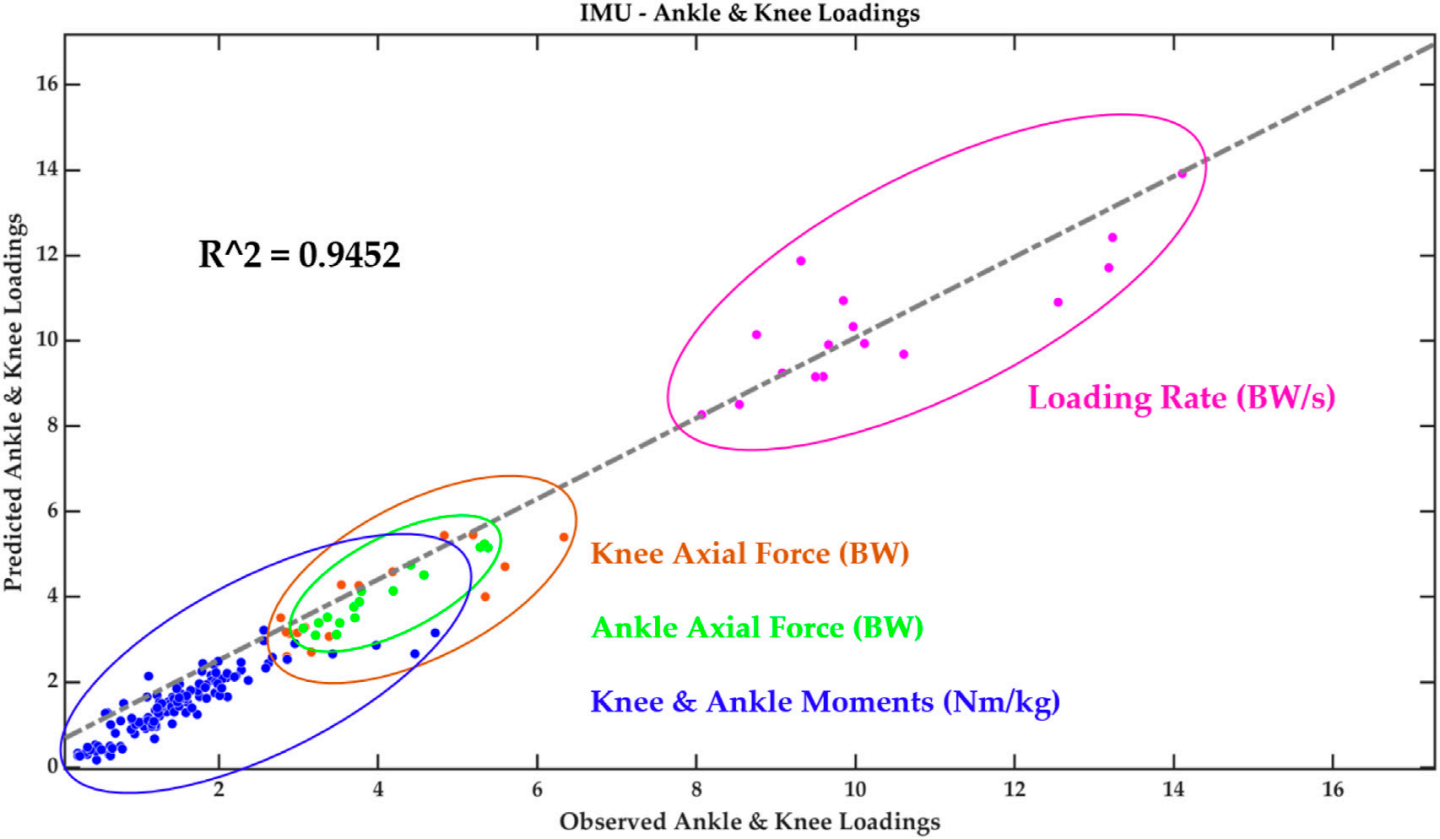
Performance and validation of the PLSR machine learning model.

Together with the four predictors, a prediction accuracy of 94.52% was observed for the moments and contact forces in the knee and ankle joints (Figure 10). To test the sensitivity of the knee and ankle peak acceleration, we performed a “leave-one-out” cross validation and found that both knee and ankle peak acceleration could predict 93.72% of the loadings. In particular, the knee peak acceleration had an 88.76% prediction accuracy and the ankle peak acceleration had a 93% prediction accuracy.

## 4 Discussion

This study integrated the wearable sensors with the in-lab motion capture analysis to correlate the wearable signals with biomechanical loading metrics and successfully implement machine learning predictive models (PCA and PLSR). The peak acceleration from wearable sensors attached to the lower limb of the badminton athletes could predict knee and ankle joint loadings with excellent accuracy (94.52%). The key findings of PCA modelling indicated that the variances between the vertical GRF of sub-maximal and maximal lunges are located in the initial and secondary impact peaks (including the loading rate region). Similar variances in the knee flexion/extension and varus/valgus moments were found within the timeframes, in addition to the axial knee contact force that mainly varied in the mid-stance. The ankle dorsi/plantar flexion and inversion/eversion moments and axial contact force showed greater variances during the initial landing (impact absorption) and push-off phases between sub-maximal and maximal lunges.

During the lab-simulated biomechanical test, we applied the well-established protocols (Kuntze et al., 2010; Huang et al., 2014; Lin et al., 2015; Lam et al., 2017; Mei et al., 2017) for motion capture and synchronously integrated the IMU sensors to set up the ground truth “gold standard” facilities. The wearable technologies have been implemented for the recognition of badminton-relevant movements for game analysis, showing promising accuracy (Steels et al., 2020). Results of stance time, approaching velocity, and joint biomechanical loadings are consistent with those of the recent studies on badminton lunge footwork (Kuntze et al., 2010; Hong et al., 2014; Huang et al., 2014; Lam et al., 2017; 2018; Chen et al., 2022). Considering the validated results, the primary applications of this study were to monitor and predict the loadings (joint moments and contact forces) in the knee and ankle joints with machine learning models.

Understanding the loading distribution and accumulation would assist the investigation of the injuries in the lower extremity of badminton players. Several recent review studies on badminton injuries and lunges (Lee and Loh, 2019; Lam et al., 2020; Phomsoupha and Laffaye, 2020) reported that fatigue of the musculature system was a key factor which contributed to reduced performance and loading accumulation (increased injury risks). Dynamic loading accumulation and distribution data in the “real-world” scenario collected from wearables during training and competition were monitored and reported based on the correlative prediction machine learning model. Typically, the results demonstrated that a combined physics-based and machine learning model offered promising solutions to tibia loading accumulation (Matijevich et al., 2020).

The main feature extracted from PCA modelling in the vertical GRF and knee and ankle moments and contact forces was the magnitude difference, considering different timeframes during stance. In particular, during the initial and secondary impact peak phases, the variances in the knee and ankle moments were observed, which may be explained by different approaching speeds between sub-maximal (∼2.5 m/s) and maximal (∼3.5 m/s) lunges, which are consistent with recent studies (Lam et al., 2018; Chen et al., 2022). The axial knee contact force varied between sub-maximal and maximal lunges during the mid-stance of the weight acceptance phase, which may be attributed to higher impact and highly activated muscular contractions (Fu et al., 2017). The difference between directional lunges was not reported as it was observed in our previous project that left-side (backhand) forward and backward lunges showed higher knee loadings than the right-side lunges (Yu et al., 2021a). This aimed to mimic the real on-court situation where shuttles were not returned in an anticipated manner from the opponent, and athletes could perform any directional lunges from unexpected scenarios.

In terms of the difference during the drive-off phase in the ankle plantar flexion moment and axial contact forces, these may show different multi-joint coordination patterns, as higher motion acceleration and deceleration could be observed in the sub-maximal and maximal lunges (Lee and Loh, 2019). Thus, the different acceleration and deceleration strategies or coordination exerted a greater impact on the ankle, which functioned as the primary interface with the court (Wei et al., 2015; Fong et al., 2021). In terms of the significant difference between the ankle and knee impact acceleration, a possible explanation could be that the tibia (shank) absorbed most of the impact at initial contact, which is considered to monitor the impact loading accumulation so as to reduce shank pain (Verrelst et al., 2014). The postural position, such as trunk bending and lumbar ratios, may also affect knee loadings, as reported in recent studies (Huang et al., 2014; Lin et al., 2015; Wang et al., 2023).

The multivariate machine learning (PLSR) model we developed showed promising performance (∼94.52% accuracy) while inputting the contact times, approaching velocity, and ankle and knee peak acceleration to predict knee and ankle loadings. These input parameters could easily be measured or calculated from IMU sensors. In order to simplify the machine learning prediction model, we trained the PLSR model with ankle and knee peak acceleration, showing similar accuracy (∼93.72%). Considering that the attachment of two belts with IMU to the proximal and distal tibiae may limit the movement of badminton athletes, we used the single knee or ankle IMU each as a predictor in the PLSR model, and the knee IMU and ankle IMU showed an accuracy of 88.76% and 93%, respectively. From the sensitivity analysis of the PLSR model we developed, it was learned that a single IMU sensor attached to the anterior distal tibia above the medial malleoli could predict approximately 93% of loadings in the ankle and knee joints, which was consistent with our clinical study (Yeung et al., 2022). Meanwhile, over 20% of total footwork measured during one single match could be used to estimate the loading accumulation in the knee and ankle joints (Valldecabres et al., 2020).

There are several limitations that should be considered before acknowledging the findings from the current study. First, the “ground-truth” synchronized data were collected in a lab-simulated court, which might not mimic the real match (or training) scenarios considering the fatigue and varied conditions. Future study shall consider a well-designed experimental setup that match the real badminton court under training and match conditions. Second, only the discrete and key datapoints were applied to train and test the intelligent statistical models, without considering the time-varying features; thus, other machine learning algorithms, deep learning algorithms, and convolutional neural networks (such as long short-term memory, LSTM) may be utilized for the monitoring and prediction of loading accumulation (Shao et al., 2022; Liew et al., 2023).

## 5 Conclusion

In summary, this study successfully utilized the wearable technology and machine learning models to predict the joint loadings in a lab-simulated badminton court test, showing promise and feasibility of application into the “real-world” scenario. The intelligent and dynamic framework developed in the current study provided a perspective to address the gap between lab and on-court analyses, taking the badminton lunge footwork as a proof-of-concept example. The intelligent monitoring and feedback of loading patterns or accumulation could be integrated to design the training and competition schemes in badminton or other court sports in a scientific manner, thus preventing fatigue, reducing potential loading-accumulation-related injury, and maximizing athletic performance.

## Data Availability

The raw data supporting the conclusion of this article will be made available by the authors, without undue reservation.
